# Efficacy and safety of leadless pacemaker implantation in octogenarians: a single-center experience

**DOI:** 10.3389/fcvm.2025.1571665

**Published:** 2025-05-07

**Authors:** Zhipeng Zhang, Zonglei Wu, Chao Luan, Shiyu Dai, FeiFei Chen, Yuchen Gao, Fenglan Huang, Yuanjun Sun, Xiaomeng Yin, Lianjun Gao, Jinqiu Liu, Xiaohong Yu

**Affiliations:** Department of Cardiology, The First Affiliated Hospital of Dalian Medical University, Dalian, Liaoning, China

**Keywords:** leadless pacemaker, octogenarians, efficacy and safety, complication, pacing induced cardiomyopathy

## Abstract

**Aims:**

Long-term complications occur in approximately 4%–12% of patients with standard transvenous pacemakers (TVPs), most of whom are elderly. The leadless pacemaker (LP) has emerged as an effective alternative to traditional TVPs, offering a lower complication profile. However, data on LP implantation in octogenarians remain limited, and concerns persist regarding its feasibility and safety in this population. This study aimed to assess the feasibility and clinical outcomes of LP implantation in octogenarians.

**Methods and Results:**

Between January 2021 and January 2024, 154 patients (mean age 75.5 ± 9.8 years) who underwent LP implantation at our center were consecutively included. The study cohort was stratified into two age groups: octogenarians (≥80 years, mean age 84.2 ± 3.3 years, *n* = 66) and non-octogenarians (<80 years, mean age 69.0 ± 7.9 years, *n* = 88). Outcomes assessed included electrical parameters, procedural characteristics, and complication rates at hospital discharge and throughout the follow-up period. The procedure was successfully performed in all patients. Six patients (five octogenarians and one non-octogenarian) underwent LP implantation along with concurrent removal of transvenous pacing systems due to prior pocket infections. No device-related infections were observed. LP implantation combined with atrioventricular node ablation (AVNA) was successfully performed in three octogenarians without any complications. Despite having a higher burden of comorbidities, as indicated by a higher age-adjusted Charlson Comorbidity Index (7 IQR 5–8 vs. 4 IQR 3–6, *p* < 0.001),the octogenarian group demonstrated comparable outcomes to the non-octogenarians. No statistically significant differences were observed between the groups regarding electrical parameters, procedure duration, fluoroscopy time during a median follow-up of 12 (IQR 6–22) months. The proportion of patients requiring more than two deployment attempts was significantly higher among octogenarians (48.5% vs. 26.1%, *p* = 0.032). These findings suggest that LP implantation is safe and effective in octogenarians when performed at experienced centers. However, the incidence of significant periprocedural complications was relatively higher in octogenarians than in non-octogenarians (4.5% vs. 1.1%), although this difference was not statistically significant.

**Conclusions:**

LP implantation appears to be a safe and effective therapeutic option for octogenarians, including those with more combabilities. Therefore, it should be considered a viable alternative to conventional TVPs in this aging population.

## 1 Introduction

The leadless pacemaker (LP) is a miniaturized pacing system that operates without the need for transvenous leads and is increasingly considered a viable alternative to single- and dual-chamber transvenous pacemakers (TVPs) in select patient populations (1–3). The LP is implanted directly into the right ventricle, eliminating the need for a subcutaneous device pocket or intravascular lead placement. Moreover, LP implantation does not require post-procedural limb immobilization, which is advantageous in elderly patients, especially those with multiple comorbidities or cognitive impairments, who cannot restrict movement independently. This feature helps reduce the risk of lead damage, lead dislodgement, and incisional infection (4, 5). Despite these advantages, several risk factors, such as low body weight, female sex, chronic obstructive pulmonary disease, and advanced age, have also been associated with procedural complications during LP implantation, similar to those observed with conventional TVP systems (6). Given the increasing demand for pacemaker therapy among elderly patients, concerns remain regarding the safety and efficacy of LP implantation in frail octogenarians with more combabilities. Therefore, further investigation into the clinical outcomes of this population is warranted. This study presents our single-center experience with LP implantation in octogenarians, focusing on procedural feasibility, safety, and clinical outcomes.

## 2 Materials and methods

### 2.1 Study population

This retrospective study included consecutive patients who underwent implantation of the Micra transcatheter pacing system (Micra TPS) at the First Affiliated Hospital of Dalian Medical University between January 2021 and January 2024 and provided informed consent. Due to the relatively high cost of LPs, patient selection was limited to individuals who had demonstrated independent mobility within their home environment prior to the onset of bradycardia. Clinical data were collected retrospectively, including patient demographics, left ventricular ejection fraction (LVEF), comorbidities, and indications for pacemaker implantation, specifically, confirmed diagnoses of bradyarrhythmia.The Charlson comorbidity index (CCI) was calculated using the method reported by Charlson, which assigns a weighted score based on the presence of certain diseases. The age-adjusted Charlson Comorbidity Index (aCCI) value was obtained through an age-adjusted calculation of the CCI index. Data regarding antiplatelet and anticoagulant use during implantation were also recorded. Procedural details such as pacing threshold, R wave amplitude, and impedance values intraoperatively and postoperatively were obtained from medical records, remote monitoring systems, and/or telephone interviews with the patient or their family members. Patients who underwent additional procedures during LP implantation, such as transvenous lead extraction or atrioventricular node ablation (AVNA), were included in the analysis. The anatomical site of device implantation was classified into one of four categories: high septum, mid septum, low septum, or apical septum based on radiographic imaging.

### 2.2 Leadless pacemaker implantation

Implantation of the Micra transcatheter pacing system (Micra TPS) was performed following the manufacturer's instructions and as previously described in the literature (7). Vascular access was obtained via the femoral vein using the Seldinger technique, followed by inserting a 27-Fr Micra™ delivery catheter into the right atrium. The delivery catheter was then advanced across the tricuspid valve into the right ventricle. The leadless pacemaker was deployed after confirming the appropriate site using right anterior oblique (RAO) and left anterior oblique (LAO) projections with contrast medium. Following deployment, a “pull and hold” test and intraoperative electrical measurements were performed to confirm appropriate positioning and function. The tether was cut once satisfactory parameters were obtained, and the delivery system was withdrawn. Hemostasis at the femoral access site was achieved using sutures placed in a configuration in Figure 8 in all patients. All patients were immobilized for at least 8 h post-implantation. Prophylactic antibiotics were administered 30 min prior to the procedure. In patients with concomitant TVP pocket infections, antibiotic therapy was typically guided by bacterial culture results and continued for 2–4 weeks following pacemaker system removal. Sutures were removed 48 h after the procedure.

### 2.3 Follow-up

Procedural data, including operative duration, number of deployment attempts, and intraoperative adverse events, and device-related parameters such as pacing capture threshold, R-wave amplitude, and pacing impedance were prospectively collected. Each patient underwent thorough evaluations to assess device function and potential adverse events at the time of hospital discharge and during the follow-up period. Follow-up visits were scheduled at 1-, 3-, 6-, and 12-months post-implantation and annually thereafter. At each visit, automated device measurements were reviewed, and the adequacy of device programming and pacing parameters was confirmed.

### 2.4 Definition of implantation complications

Significant complications were categorized as either peri-procedural or follow-up complications. Peri-procedural complications were defined according to established literature. They included procedure-related death within 30 days, permanent loss of device function, pericardial effusion (with or without the need for interventional or surgical management), device revision within 30 days, infection, device dislodgement, severe tricuspid valve damage, and femoral artery injury or hematoma requiring intervention. Follow-up complications included events such as loss of pacing capture, elevated pacing thresholds, pacemaker syndrome, pacing-induced cardiomyopathy (PICM), and worsening of tricuspid regurgitation (TR) or mitral regurgitation(MR). A high pacing threshold was defined as a capture threshold exceeding 1.0 V at 0.24 ms pulse width.

### 2.5 Statistical analysis

Demographic, procedural, and outcome data were extracted from electronic medical records. Categorical variables are presented as frequencies and percentages, while continuous variables are expressed as means with standard deviations (SDs) or medians with 25th–75th percentiles (interquartile range) based on distribution. The Student's *T*-test, or non-parametric equivalent Mann–Whitney was used for comparison of continuous variables. Categorical variables were compared using the chi-square (*χ*^2^) test. Univariate and multivariate logistic regression analyses were performed to identify risk factors associated with significant complications.Variables with a *p*-value < 0.1 at univariate analysis were enrolled in multivariate analysis. A two-tailed *p*-value < 0.05 was considered statistically significant. All statistical analyses were conducted using the SPSS software package (IBM SPSS Statistics, version 27.0).

## 3 Results

### 3.1 Study population and clinical characteristics

General information of the study population is presented in Table 1. A total of 154 patients were included in the analysis, with a median follow-up duration of 12 (IQR 6–22) months. The mean age was 75.5 ± 9.8 years, and 63% of the cohort were male. Regarding comorbid conditions, coronary artery disease was present in 26.6% of patients, hypertension in 63.6%, and AF in 68 patients (44.2%). Among those with AF, 63 patients (92.6%) were receiving oral anticoagulation therapy. The average left ventricular ejection fraction (LVEF) was 57 ± 6%. The primary indications for pacemaker implantation were AF with a slow ventricular response (29.9%) and atrioventricular (AV) block (35.1%). Sixty-six patients (42.9%) were aged ≥80 years and were categorized as octogenarians, with a mean age of 84.2 ± 3.3 years. Compared to non-octogenarians, the octogenarian group had a significantly higher prevalence of heart failure (42.4% vs. 26.1%, *p* = 0.039), chronic renal failure (20% vs. 5.7%, *p* = 0.010), and AF (54.5% vs. 36.4%, *p* = 0.033). The aCCI of octogenarians was significant higher than no-octogenarians(7 IQR 3–6 vs. 4 IQR 5–8, *p* < 0.001). and the percentage of aCCI ≥ 5 is 84.8% in octogenarians and only 43.2% in non- octogenarians (*p* < 0.001). No other significant differences in baseline characteristics were observed between the two groups. A total of 18 patients in the study were identified as having renal insufficiency, of whom 5 had an estimated glomerular filtration rate (eGFR) below 30 ml/min and 3 required maintenance hemodialysis. No statistically significant difference was observed between preoperative and discharge serum creatinine levels in this subgroup (164 IQR 152–205 µmol/L vs. 160 IQR 144–177 µmol/L, *p* = 0.203).

**Table 1:** Baseline characteristics.

Characteristics	Total patients *n* = 154	Nonoctogenarians *n* = 88	Octogenarians *n* = 66	*P*-value
Age, mea*n* ± SD, years	75.5 ± 9.8	69.0 ± 7.9	84.2 ± 3.3	< 0.001
Male (n, %)	97 (63.0)	52 (59.1)	45 (68.2)	0.248
LVEF %, mean ± SD	57.0 ± 6.0	57.7 ± 5.6	56.1 ± 6.5	0.091
Comorbidities				
Coronary artery disease (n, %)	41 (26.6)	19 (21.6)	22 (33.3)	0.140
Hypertension (n, %)	98 (63.6)	52 (59.1)	46 (69.7)	0.236
Valvular disease (n, %)	35 (22.7)	20 (22.7)	15 (22.7)	1.000
Heart failure (n, %)	51 (33.1)	23 (26.1)	28 (42.4)	0.039
Chronic renal failure (n, %)	18 (11.8)	5 (5.7)	13 (20.0)	0.010
eGFR < 30 ml/min(1.73m^2^) (n, %)	5 (3.2)	2 (2.3)	3 (4.5)	0.652
COPD (n, %)	10 (6.5)	4 (4.5)	6 (9.1)	0.328
Diabetes mellitus (n, %)	48 (31.2)	28 (31.8)	20 (30.3)	0.862
Stroke (n, %)	17 (11)	6 (6.8)	11 (16.7)	0.070
Cancer (n, %)	7 (4.5)	2 (2.3)	5 (7.6)	0.139
Atrial fibrillation (n, %)	68 (44.2)	32 (36.4)	36 (54.5)	0.033
aCCI	5 (IQR 3–7)	4 (IQR 3–6)	7 (IQR 5–8)	<0.001
aCCI ≥ 5 (n, %)	94 (61.0)	38 (43.2)	56 (84.8)	<0.001
Anticoagulation therapy (n, %)	63 (40.9)	31 (35.2)	32 (48.5)	0.102
Anti-platelet therapy (n, %)	72 (46.8)	45 (51.1)	27 (40.9)	0.208
Pacing indication				
Slow AF (n, %)	46 (29.9)	24 (27.3)	22 (33.3)	0.478
AF and AV block (n, %)	9 (5.8)	3 (3.4)	6 (9.1)	0.173
AV block (n, %)	54 (35.1)	35 (39.8)	19 (28.8)	0.175
Tachy-Brady syndrome (n, %)	7 (4.5)	5 (5.7)	2 (3.0)	0.699
Sinus node dysfunction (n, %)	27 (17.5)	19 (21.6)	8 (12.1)	0.140
Pocket infection (n, %)	6 (3.9)	4 (6.1)	2 (2.3)	0.701
Leads fracture/perforation (n, %)	2 (1.3)	0	2 (3.0)	0.182
AVNA (n, %)	3 (1.9)	0	3 (4.5)	0.077

Values are expressed as mean ± SD, median (IQR), or *n* (%).

aCCI, age-adjusted Charlson Comorbidity Index; AF, atrial fibrillation; AV atrioventricular; AVNA, atrioventricular node ablation; COPD, chronic obstructive pulmonary disease; eGFR, estimated glomerular filtration rate; IQR, interquartile range; LVEF, left ventricular ejection fraction; SD, standard deviation.

Among the 154 patients, 35 (22.7%) had a history of cardiac valvular disease, with 11 (31.4%) having undergone prior valve interventions. These included 2 patients with post-transcatheter aortic valve implantation, 2 with isolated aortic valve replacement, one with isolated mitral valve replacement, one with isolated tricuspid valvuloplasty, and 5 with multiple valve surgeries.

Additionally, six patients (5 in octogenarian, 1 in non-octogenarian) received a LP due to prior device-related pocket infections, and underwent concurrent transvenous lead extraction and pocket debridement. Three octogenarians with fast AF underwent simultaneous AVNA during LP implantation.

Three octogenarian patients presented with rapid AF and heart failure with preserved or mildly reduced ejection fraction. They experienced hemodynamic instability during AF episodes and were refractory to pharmacological treatment, with symptoms corresponding to New York Heart Association (NYHA) Class III–IV. Comorbidities included hypertension, hypothyroidism, COVID-19-associated pneumonia, coronary artery disease, and old myocardial infarction. All three patients underwent LP(Micra) implantation combined with AVNA. The combined procedure durations were approximately 50, 40, and 45 min. Following the intervention, their heart function improved to NYHA Class I–II, and no hospitalizations were recorded during the 6- to 48-month follow-up period. Notably, no similar cases were observed in the non-octogenarian group.

### 3.2 Procedure details

**Figure 1:**
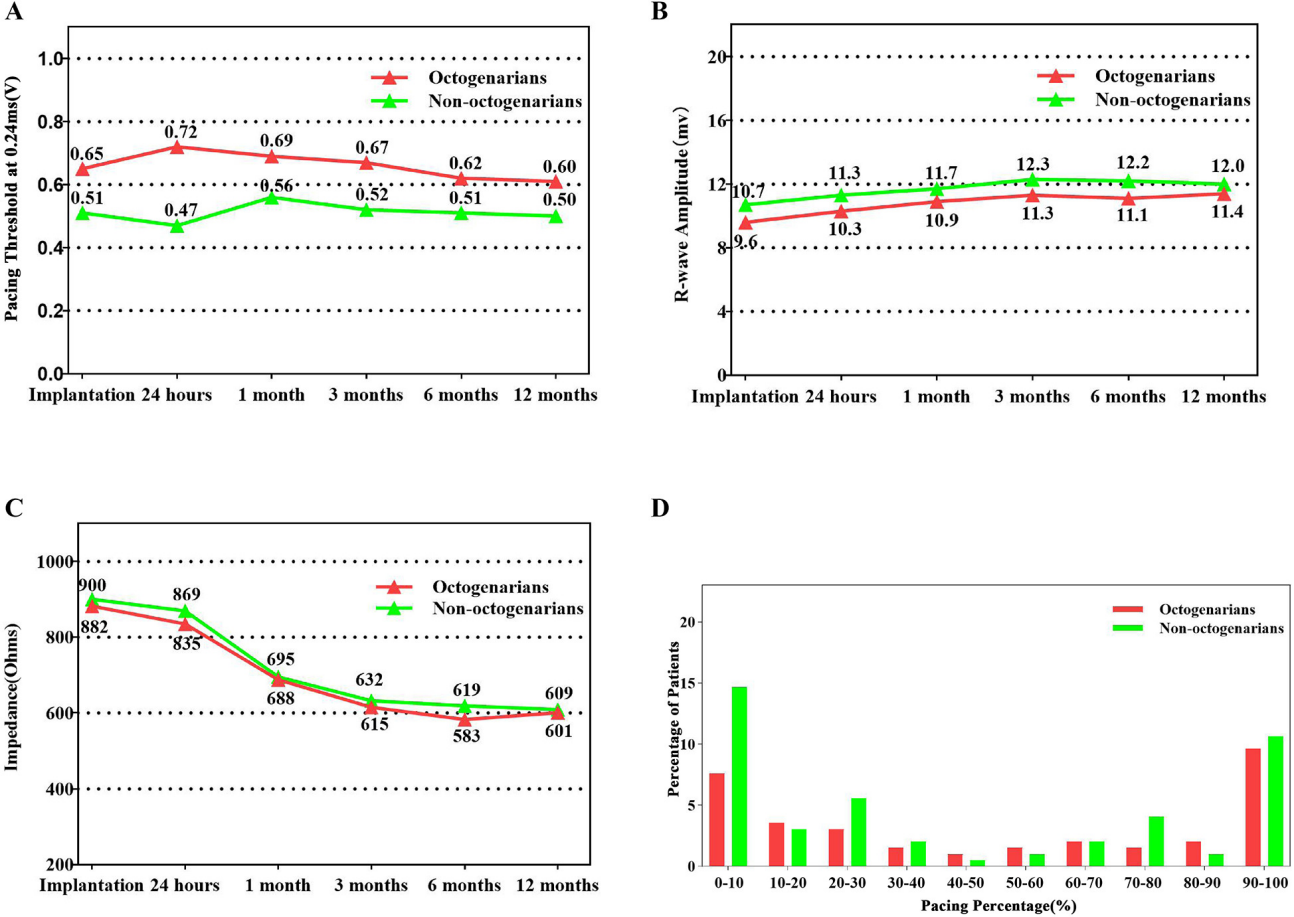
Summary of device electrical parameters and ventricular pacing percentage. **(A)** Average pacing capture threshold at 0.24 ms over time. **(B)** Average R-wave sensing amplitude over time. **(C)** Average pacing impedance over time. **(D)** Distribution of ventricular pacing percentage at last device interrogation.

Detailed procedural data are summarized in Table 2. All LP implantations were performed under fluoroscopic guidance in a cardiovascular catheterization laboratory. The mean procedural duration was 47.4 ± 8.7 min. Electrical performance parameters at implantation included an average sensing amplitude of 10.3 ± 4.7 mV, impedance of 892 ± 294 Ohms, and a pacing threshold of 0.51 ± 0.38 V at a pulse width of 0.24 ms. The percentage of ventricular pacing of octogenarians was comparable to non-octogenarians(45.7% vs. 27.3%, *p* = 0.108). The radiographic assessment revealed that the LP was most commonly implanted in the lower-mid septal region of the right ventricle, accounting for 85.6% of cases. There were no statistically significant differences between octogenarians and non-octogenarians regarding implant location, electrical parameters, procedure duration, or fluoroscopic time. These parameters remained stable throughout the follow-up period (Figure 1). However, the proportion of patients requiring more than two deployment attempts was significantly higher among octogenarians (48.5% vs. 26.1%, *p* = 0.032). Of the total cohort, 34 patients (22.1%) with high-degree atrioventricular (AV) block, including 15 octogenarians (44.1%) and 19 non-octogenarians (55.9%), received a Micra AV to enable AV-synchronized ventricular pacing. The overall mean AV synchrony was 84.8 ± 12.1%. Following programming optimization, AV synchrony in octogenarians was comparable to non-octogenarians (85.2 ± 13.3% vs. 84.0 ± 11.1%, *p* = 0.879).

**Table 2:** Procedure details.

Characteristics	Total patients *n* = 154	Nonoctogenarians *n* = 88	Octogenarians *n* = 66	*P*-value
Procedure time (min)	47.4 ± 8.7	46.8 ± 7.9	48.2 ± 8.3	0.301
Radiation time (min)	5.1 ± 1.2	5.0 ± 1.4	5.2 ± 1.1	0.177
Deployment attempts (n, %)				
1	99 (64.2)	65 (73.9)	34 (51.5)	0.032
2	32 (20.8)	12 (13.6)	20 (30.3)	
3	17 (11.0)	8 (9.1)	9 (13.6)	
4	6 (3.9)	3 (3.4)	3 (4.5)	
R wave amplitude (mv)	10.3 ± 4.7	10.7 ± 4.9	9.6 ± 4.4	0.157
Pacing threshold (V)	0.51 ± 0.38	0.53 ± 0.28	0.47 ± 0.22	0.152
Impedance (*Ω*)	892 ± 294	900 ± 278	882 ± 317	0.707
Location of the device (n, %)				
High-septum of right ventricular	15 (9.7)	12 (13.6)	3 (4.5)	0.100
Mid-septum of right ventricular	85 (55.1)	42 (47.7)	43 (65.2)	
Lower-septum of right ventricular	47 (30.5)	29 (33.0)	18 (27.3)	
Apical-septum of right ventricular	7 (4.5)	5 (5.7)	2 (3.0)	
Ventricular pacing percentage (%)	32.3 (IQR 7.0–90.0)	27.3 (IQR 4.2–87.1)	45.7 (IQR 11.0–95.0)	0.108
AV mean synchronized percentage (%)	84.8 ± 12.1 (*n* = 34)	84.0 ± 11.1 (*n* = 19)	85.2 ± 13.3 (*n* = 15)	0.879

Values are expressed as mean ± SD, median (IQR), or *n* (%).

AV, atrioventricular; IQR, interquartile range; SD, standard deviation.

### 3.3 Perioperative and follow-up complication rates

Major periprocedural and follow-up complications are summarized in Table 3. A total of 18 complications (11.7%) were observed in 14 patients: 10 complications occurred in the octogenarian group and 8 in the non-octogenarian group. Aside from 3 cases of pericardial effusion (1.9%) and one case of deep vein thrombosis, no other periprocedural complications were detected. However, one case of open-chest operation due to cardiac perforation and the only one case of deep vein thrombosis occurred in octogenarian group. Thirteen follow-up complications were recorded, comprising one case of elevated pacing threshold (1.63 V at 0.24 ms), 7 cases of worsening TR and MR, and 5 cases of pacemaker syndrome. Among the latter, 4 patients demonstrated mild declines in LVEF during follow-up and were classified as PICM.

**Table 3:** Major complications for patients during peri-procedure and follow-up.

Characteristics	Total patients *n* = 154	Nonoctogenarians *n* = 88	Octogenarians *n* = 66	*P*-value
Peri-procedure complications (n, %)	4 (2.6)	1 (1.1)	3 (4.5)	0.314
Pericardial effusion (n, %)	3 (1.9)	1 (1.1)	2 (3.0)	0.577
Device dislodgement (n, %)	0	0	0	NA
Procedure-related death (n, %)	0	0	0	NA
Device infection (n, %)	0	0	0	NA
Deep vein thrombosis (n, %)	1 (0.6)	0	1 (1.5)	0.429
Pulmonary embolism (n, %)	0	0	0	NA
Acute kidney injury (n, %)	0	0	0	NA
Puncture site infection (n, %)	0	0	0	NA
Puncture site haematoma (n, %)	0	0	0	NA
Femoral artery injury (n, %)	0	0	0	NA
Follow-up complications (n, %)	14 (9.1)	7 (8.0)	7 (10.6)	0.584
Eelevated thresholds (n, %)	1 (0.6)	0	1 (1.5)	0.429
Worsening of TR (n, %)	5 (3.2)	3 (3.4)	2 (3.0)	1.000
Worsening of MR (n, %)	2 (1.3)	2 (2.3)	0	0.507
Pacemaker syndrome (n, %)	5 (3.2)	2 (2.3)	3 (4.5)	0.652
PICM (n, %)	4 (2.6)	2 (2.3)	2 (3.0)	1.000
Device upgrade (n, %)	1 (0.6)	0	1 (1.5)	0.429
Total events (n, %)	18 (11.7)	8 (9.1)	10 (15.2)	0.247

Values are expressed as *n* (%).

MR, mitral regurgitation; NA, not applicable; PICM, pacing induced cardiomyopathy; TR, tricuspid regurgitation.

**Figure 2:**
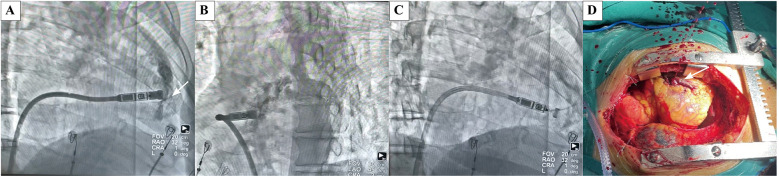
Case of an octogenarian who developed acute cardiac tamponade during the procedure and subsequently underwent emergency open chest surgery. **(A)** Extravasation of contrast agent into the pericardial space following contrast agent injection in RAO(white arrow indicates the perforation site). **(B)** Extravasation of contrast agent into the pericardial space following contrast agent injection in LAO. **(C)** Extravasation of contrast agent into the pericardial space following contrast agent injection in RAO, there are amounts of excess fluid around the heart. **(D)** Perforation caused by cutting of the delivery catheter was located at the anterior interventricular sulcus, between the right ventricular free wall and septum at open chest surgery. (white arrow indicates the perforation site).

The clinical presentations of the 3 cases of pericardial effusion are described below:

Case 1: An 84-year-old male diagnosed with second-degree AV block (2:1 conduction) and a heart rate of 30–42 bpm for three months was scheduled for Micra VR implantation. The patient had experienced thoracoscopic lobectomy, hypertension, diabetes mellitus, heart failure, COPD, chronic kidney disease (eGFR: 29 ml/min; creatinine: 181 µmol/L), and moderate anemia. Pericardial effusion was detected immediately following the injection of contrast medium, prior to device implantation (Figures 2A,B). LP was implanted at the mid-septum with another deployment. Electrical parameters were optimal (Figure 2C). Emergent pericardial drainage yielded over 800 ml blood, and the blood pressure dropped to 80/60 mmHg. Emergency thoracotomy revealed myocardial perforation at the anterior interventricular sulcus, located between the right ventricular free wall and septum, attributed to trauma by the tip of the delivery catheter (Figure 2D). The patient was transferred to the intensive care unit for three days and was discharged two weeks later without residual complications.
Case 2: An 88-year-old male with second-degree AV block (2:1 conduction), with a recent recovery from COVID-19 pneumonia, chronic renal failure (eGFR: 21 ml/min; creatinine: 253 µmol/L). Shortly after implantation, the patient developed nausea, diaphoresis and hypotension (76/42 mmHg). The pericardiocentesis yielded approximately 200 ml of blood. The patient was discharged without complications five days later.
Case 3: A 67-year-old female with paroxysmal AF and prolonged pauses (>5 s) with hypertension, hypothyroidism, and atrial septal defect repairment. The less pericardial effusion without hemodynamic compromise at four hours post-implantation, which resolved spontaneously within seven days.

Risk factors for complications were evaluated using univariate and multivariate logistic regression analyses. Two independent predictors were identified: a higher aCCI (OR = 1.234, 95% CI: 1.028–1.483, *p* = 0.024) and the need for three or more deployment attempts during implantation (OR = 5.287, 95% CI: 1.259–22.203, *p* = 0.023) (Table 4).

**Table 4:** Univariable and multivariable logistic regression analysis.

Characteristics	Univariate analysis		Multivariate analysis	
	OR (95% CI)	*P*-value	OR (95% CI)	*P-*value
Age, years	1.018 (0.959–1.081)	0.559		
Male	1.064 (0.338–3.345)	0.996		
LVEF,%	0.985 (0.901–1.076)	0.732		
Coronary artery disease	0.192 (0.024–1.519)	0.118		
Hypertension	1.477 (0.441–4.950)	0.527		
Valvular disease	0.920 (0.242–3.502)	0.903		
Heart failure	1.135 (0.360–3.581)	0.829		
Chronic renal failure	3.571 (0.989–12.902)	0.052	2.374 (0.548–10.284)	0.248
COPD	2.750 (0.524–14.439)	0.232		
Diabetes mellitus	0.576 (0.513–2.166)	0.414		
Cancer	1.718 (0.192–15.384)	0.629		
AF	2.471 (0.788–7.754)	0.121		
aCCI	1.260 (1.071–1.482)	0.005	1.234 (1.028–1.483)	0.024
Procedure time, min	1.003 (0.990–1.015)	0.661		
deployment times ≥ 3	5.584 (1.510–20.656)	0.010	5.287 (1.259–22.203)	0.023
Pacing QRS duration, ms	0.970 (0.932–1.009)	0.134		
Pacing percent,%	1.000 (0.995–1.006)	0.872		
R wave amplitude,mv	1.038 (0.934–1.154)	0.490		
Pacing threshold, V	0.705 (0.067–7.459)	0.705		
Impedance, *Ω*	0.997 (0.993–1.002)	0.267		
High-septum location	2.081 (0.248–17.430)	0.303		

Characteristics of *p* < 0.1 in univariable analysis were enrolled in multivariable analysis.

aCCI, age-adjusted Charlson comorbidity indeX; AF, atrial fibrillation; CI, confidence interval; COPD, chronic obstructive pulmonary disease; LVEF, left ventricular ejection fraction; OR, odds ratio.

## 4 Discussion

This study enrolled 154 consecutive patients, including 66 octogenarians, who underwent LP implantation. The findings suggest LP implantation in octogenarians is safe and effective when performed at experienced centers. However, the incidence of significant periprocedural complications was relatively higher among octogenarians than non-octogenarians (4.5% vs. 1.1%). This may be attributable to the increased number of deployment attempts in the octogenarian group (48.5% vs. 26.1%, *p* = 0.032), as well as a more significant comorbidity burden, as reflected by a higher aCCI (7 IQR 3–6 vs. 4 IQR 5–8, *p* < 0.001).

### 4.1 General comparison between octogenarians and non-octogenarians

This study is a retrospective, single-center analysis suggesting that LP implantation in octogenarians is as feasible as in non-octogenarians.

The primary indications for pacing were high-degree atrioventricular (AV) block (*n* = 54, 35.1%) and AF (*n* = 46, 29.9%). Additionally, 17.5% of the overall population underwent LP implantation due to sinus bradycardia or episodes of prolonged pauses. The Micra AV device was implanted in 15 octogenarians and 19 non-octogenarians. The atrioventricular-synchronized pacing percentage was comparable between the two groups (85.2% ± 13.3% vs. 84.0% ± 11.1%, *p* = 0.879).

Device parameters remained stable over time in both groups during follow-up, with the exception of one octogenarian patient in whom an increase in pacing threshold was observed from 1.25 V/0.24 ms to 1.63 V/0.24 ms at the 6-month follow-up. The threshold remained stable thereafter. No patients experienced device dislodgement. Moreover, there were no significant differences in procedure duration or radiation exposure time between the groups, contrasting with findings reported in previous studies (8). In our center, the device was implanted in the right ventricle's lower-mid septum in most patients (85.6%). The mean sensing amplitude was 10.3 ± 4.7 mV, the mean impedance was 892 ± 294 Ohm, and the mean pacing threshold was 0.51 ± 0.38 V at 0.24 ms. Optimal electrical parameters may be associated with favourable prognosis.

### 4.2 Complications of leadless pacemaker implantation

Pericardial effusion occurred early in the operator's learning curve. One octogenarian with cardiac tamponade required emergency open-chest surgery, during which 800 ml of bloody pericardial fluid was drained. The perforation was located at the anterior interventricular sulcus, between the right ventricular free wall and septum, and was attributed to the sharp tip of the delivery catheter. This case underscores the importance of gentle manipulation of the delivery system, especially during traversal of the tricuspid valve, and avoiding forceful advancement toward the target site before contrast injection to prevent cardiac perforation. Implanting LP in the septum rather than the apex may help reduce the risk of cardiac perforation. However, differentiating between septal and anterior interventricular sulcus positioning based solely on contrast radiography can be challenging. A relatively sparse trabecular pattern in the RAO view may indicate placement near the anterior interventricular sulcus. Notably, LP implantation in elderly patients may demand technical expertise due to altered cardiac positioning resulting from scoliosis, prior pulmonary lobectomy, atelectasis, or other thoracic deformities. The incidence of pericardial effusion increases significantly with more deployment attempts (9).

Risk factors for pericardial effusion during LP implantation are similar to those associated with traditional TVPs, including advanced age, BMI < 20, female sex, heart failure, prior myocardial infarction, COPD, absence of prior cardiothoracic surgery, and dialysis. Both perioperative and postoperative complications remain a concern in octogenarians. In our study, significant complications of cardiac perforation was 3.0% in octogenarians, compared to 1.1% in non-octogenarians. The reported rates of cardiac perforation or pericardial effusion ranging from 1.1% to 8% (2).

Worsening TR was observed in five patients (3.2%) during follow-up with no statistic significant between two groups. 80% (4/5) of these cases occurred within the first 30 implantations. The impact of LP implantation on cardiac structure and valvular function remains uncertain. Beurskens et al. reported a correlation between higher septal positioning and worsening TR (10). Hai et al. identified the distance between the LP tip and the tricuspid annulus as the only independent predictor of TR (11). These findings suggest that operators should avoid high septal deployment and carefully consider the spatial relationship between the LP tip and the atrioventricular valve annulus. Moreover, gentle catheter manipulation and minimizing deployment attempts may help mitigate the risk of TR.

### 4.3 Leadless pacemaker and infections

No device-related infections were observed in our cohort, aligning with findings from the IDE and PAR studies (1, 2). Infection-related comorbidities are more common in elderly individuals, particularly those who are frail or have underlying conditions such as renal insufficiency, diabetes mellitus, or a history of valvular interventions. In such cases, LP implantation may represent an optimal option for permanent pacing in older patients (12). In our study, six patients received LP implantation and concurrent transvenous pacing system remove due to pocket infections, five octogenarians. After replacing infected cardiac implantable electronic devices (CIEDs) with LPs, no reinfections were reported during the follow-up period. These findings support existing evidence that LPs offer a safe pacing alternative for patients with prior CIED infections who require device extraction (13). Octogenarians represent a vulnerable population with a high comorbidity burden, often unable to tolerate prolonged procedures and more susceptible to infections. In this context, LPs offer advantages over TVPs. Although LPs are more expensive than TVPs, their cost-effectiveness in elderly patients should not be underestimated.

### 4.4 Simultaneously leadless pacemaker implantation and AVNA

LP implantation combined with AVNA was successfully performed in three octogenarians in our study without any complications. These patients presented with AF that responded poorly to pharmacological or procedural rate-control strategies, necessitating LP implantation and concurrent AVNA. The average duration of the combined procedures was 45 min. Postoperatively, all three patients experienced improvement in heart function, and no hospitalizations for heart failure occurred during the follow-up period. Simultaneous LP implantation and AVNA through a single femoral venous puncture offer the advantage of completing both procedures in one session. The short procedural time benefits elderly patients with more severe symptoms and multiple comorbidities. This approach may offer additional clinical benefits compared TVP implantation combined with AVNA. A multicenter prospective observational study has demonstrated the safety and feasibility of LP implantation in patients undergoing AVNA for AF (14). However, potential risks associated with simultaneous AVNA and LP implantation must be considered. Implanting the LP in the mid-to-lower interventricular septum may help reduce mechanical, electrical, and thermal complications during AVNA (15). Importantly, a safe distance of at least 35 mm between the ablation catheter and the Micra device has been recommended to avoid acute pacing threshold elevation and associated risks (16).

### 4.5 Leadless pacemaker implantation and PICM

Five patients (3 octogenarians) in our study developed PICM, presenting with moderately reduced LVEF. One patient in the octogenarian group underwent a device upgrade to conduction system pacing, resulting in LVEF recovery above 50%. The remaining four patients achieved LVEF improvement to over 50% with guideline-directed medical therapy for heart failure. The percentage of ventricular pacing in patients with high-degree AV block who developed PICM ranged from 80% to 100%. Four of these five cases had been implanted with the Micra VR device. LPs are associated with a lower incidence of PICM than TVPs. In a study by Sanchez et al, PICM occurred in 13.7% (18/131) of patients in the TVP group vs. only 3% (2/67) in the LP group (17). Additionally, the anatomical location of LP implantation plays a role in PICM risk. Devices implanted in the apical or apical septal region were associated with a higher incidence of PICM compared to those positioned in the mid or high septum (18). These findings underscore the importance of optimal device selection and implantation site to reduce the risk of PICM.

In summary, compared to the non-octogenarian group, octogenarians exhibited a higher burden of comorbidities. One case of cardiac perforation requiring open-chest surgery occurred in the octogenarian group. Five octogenarians underwent LP implantation in combination with removing infected transvenous pacing systems or AVNA. LPs were successfully implanted in all patients, with short procedure times—even in combined procedures. Device parameters remained stable throughout follow-up, with no device infection or dislodgement.

Bradycardia is a life-threatening condition that often requires emergency pacemaker implantation. Although generally considered a low-risk procedure, complication rates range from 3.8% to 12.4% and approximately 7.4% in the elderly population. Moreover, even nonagenarians with severe bradyarrhythmia can achieve comparable life expectancies with TVPs (19). LPs have emerged as an effective alternative to TVPs (20), including for octogenarians with small body habitus or cognitive impairment (21). Thus, extremely old age alone should not be considered a barrier to pacemaker implantation with indication.

Elderly patients are often frail and less tolerant of surgical procedures and pharmacological treatments. A higher burden of comorbidities usually requires more complex clinical management, presenting challenges for physicians and patients. Our study suggests that LPs offer advantages in short procedure times—even in combined interventions. However, the sharp tip of the large delivery sheath and risk of cardiac perforation must be carefully considered. From our perspective, elderly candidates should have demonstrated independent mobility before the onset of bradycardia. Clinical decision-making should be individualized, considering life expectancy, quality of life, cost-effectiveness, and the patient's overall clinical condition.

## 5 Conclusion

Our retrospective study demonstrated that LPs were successfully implanted in octogenarians with a low incidence of complications, even when combined procedures were performed simultaneously. There were no significant differences in device efficacy, safety, or performance between octogenarians and non-octogenarians. Careful catheter manipulation and minimizing the number of deployment attempts may help reduce the risk of cardiac perforation.

## 6 Limitations

The limitations of our study include its retrospective and single-center design, as well as the relatively small sample size. These factors may introduce selection bias, as the operator was the sole decision-maker regarding LP implantation and the complication rates. These limitations should be taken into account when interpreting the results. Therefore, prospective studies with larger sample sizes are necessary to further validate our findings. Additionally, due to the initial lack of a quantitative assessment of patients' frailty status, we were unable to fully investigate the relationship between frailty and study outcomes. Nevertheless, despite these constraints, our study provides valuable insights into real-world, single-center experience.

## Data Availability

The original contributions presented in the study are included in the article/Supplementary Material, further inquiries can be directed to the corresponding authors.

## References

[1] El-Chami MF Al-Samadi F Clementy N Garweg C Martinez-Sande JL Piccini JP Updated performance of the Micra transcatheter pacemaker in the real-world setting: a comparison to the investigational study and a transvenous historical control. Heart Rhythm. (2018) 15:1800–7. 10.1016/j.hrthm.2018.08.005 30103071

[2] El-Chami MF Garweg C Clementy N Al-Samadi F Iacopino S Martinez-Sande JL Leadless pacemakers at 5-year follow-up: the Micra transcatheter pacing system post-approval registry. Eur Heart J. (2024) 45:1241–51. 10.1093/eurheartj/ehae101 38426911 PMC10998730

[3] Oliveira VMR Rivera A Oliveira IC de Sousa AM Nishikubo MEP Serpa F The effectiveness and safety of leadless pacemakers: an updated meta-analysis. Curr Cardiol Rep. (2024) 26:789–99. 10.1007/s11886-024-02079-6 38869811

[4] Reynolds D Duray GZ Omar R Soejima K Neuzil P Zhang S A leadless intracardiac transcatheter pacing system. N Engl J Med. (2016) 374:533–41. 10.1056/NEJMoa1511643 26551877

[5] Ritter P Duray GZ Zhang S Narasimhan C Soejima K Omar R The rationale and design of the Micra transcatheter pacing study: safety and efficacy of a novel miniaturized pacemaker. Europace. (2015) 17:807–13. 10.1093/europace/euv026 25855677

[6] Steinwender C Khelae SK Garweg C Chan JYS Ritter P Johansen JB Atrioventricular synchronous pacing using a leadless ventricular pacemaker: results from the MARVEL 2 study. JACC Clin Electrophysiol. (2020) 6:94–106. 10.1016/j.jacep.2019.10.017 31709982

[7] Roberts PR Clementy N Al Samadi F Garweg C Martinez-Sande JL Iacopino S A leadless pacemaker in the real-world setting: the Micra transcatheter pacing system post-approval registry. Heart Rhythm. (2017) 14:1375–9. 10.1016/j.hrthm.2017.05.017 28502871

[8] Hofer D Regoli F Saguner AM Conte G Jelisejevas J Caputo ML Efficacy and safety of leadless pacemaker implantation in octogenarians. Cardiology. (2023) 148:441–7. 10.1159/000532075 37487479

[9] Piccini JP Cunnane R Steffel J El-Chami MF Reynolds D Roberts PR Development and validation of a risk score for predicting pericardial effusion in patients undergoing leadless pacemaker implantation: experience with the Micra transcatheter pacemaker. Europace. (2022) 24:1119–26. 10.1093/europace/euab315 35025987 PMC9301971

[10] Beurskens NEG Tjong FVY de Bruin-Bon RHA Dasselaar KJ Kuijt WJ Wilde AAM Impact of leadless pacemaker therapy on cardiac and atrioventricular valve function through 12 months of follow-up. Circ Arrhythm Electrophysiol. (2019) 12:e007124. 10.1161/CIRCEP.118.007124 31060371

[11] Hai JJ Mao Y Zhen Z Fang J Wong CK Siu CW Close proximity of leadless pacemaker to tricuspid Annulus predicts worse tricuspid regurgitation following septal implantation. Circ Arrhythm Electrophysiol. (2021) 14:e009530. 10.1161/CIRCEP.120.009530 33993700

[12] Garweg C Vandenberk B Foulon S Poels P Haemers P Ector J Leadless pacemaker for patients following cardiac valve intervention. Arch Cardiovasc Dis. (2020) 113:772–9. 10.1016/j.acvd.2020.05.012 32891563

[13] El-Chami MF Johansen JB Zaidi A Faerestrand S Reynolds D Garcia-Seara J Leadless pacemaker implant in patients with pre-existing infections: results from the Micra postapproval registry. J Cardiovasc Electrophysiol. (2019) 4:569–74. 10.1111/jce.13851 30661279 PMC6850680

[14] Yarlagadda B Turagam MK Dar T Janagam P Veerapaneni V Atkins D Safety and feasibility of leadless pacemaker in patients undergoing atrioventricular node ablation for atrial fibrillation. Heart Rhythm. (2018) 15:994–1000. 10.1016/j.hrthm.2018.02.025 29496606

[15] Ho J Prutkin JM. Simultaneous atrioventricular node ablation and leadless pacemaker implantation. HeartRhythm Case Rep. (2017) 3:186–8. 10.1016/j.hrcr.2016.12.007 28491798 PMC5420079

[16] Racine HP Dognin N Zhao Y Plourde B Steinberg C Whitman T Acute pacing threshold elevation during simultaneous Micra leadless pacemaker implantation and AV node ablation: clinical cases, computer model and practical recommendations. Pacing Clin Electrophysiol. (2023) 10:1269–77. 10.1111/pace.14814 37664970

[17] Sanchez R Nadkarni A Buck B Daoud G Koppert T Okabe T Incidence of pacing-induced cardiomyopathy in pacemaker-dependent patients is lower with leadless pacemakers compared to transvenous pacemakers. J Cardiovasc Electrophysiol. (2021) 32:477–83. 10.1111/jce.14814 33205561 PMC7984287

[18] Shantha G Brock J Singleton M Kozak P Bodziock G Bradford N Anatomical location of leadless pacemaker and the risk of pacing-induced cardiomyopathy. J Cardiovasc Electrophysiol. (2023) 34:1418–26. 10.1111/jce.15925 37161942

[19] Chao TF Liu CJ Tuan TC Liao JN Lin YJ Chen TJ Long-term prognosis of patients older than ninety years after permanent pacemaker implantation: does the procedure save the patients? Can J Cardiol. (2014) 10:1196–201. 10.1016/j.cjca.2014.04.010 25262861

[20] Pagan E Gabriels J Khodak A Chang D Beldner S Epstein LM Safety of leadless pacemaker implantation in the very elderly. Heart Rhythm. (2020) 12:2023–8. 10.1016/j.hrthm.2020.05.022 32454218

[21] Tachibana M Banba K Matsumoto K Ohara M. The feasibility of leadless pacemaker implantation for superelderly patients. Pacing Clin Electrophysiol. (2020) 43:374–81. 10.1111/pace.13894 32134134

